# Faunistic Composition, Ecological Properties, and Zoogeographical Composition of the Elateridae (Coleoptera) Family in the Western Black Sea Region of Turkey

**DOI:** 10.1673/031.013.14401

**Published:** 2013-12-07

**Authors:** Mahmut Kabalak, Osman Sert

**Affiliations:** Hacettepe University Faculty of Science Department of Biology 06800 Beytepe Ankara Turkey

**Keywords:** abundance, click beetles, distribution, habitat preference, phenology, species composition

## Abstract

The main aim of this study was to understand the faunistic composition, ecological properties, and zoogeographical composition of the family Elateridae (Coleoptera) of the Western Black Sea region of Turkey. As a result, 44 species belonging to 5 subfamilies and 19 genera were identified. After adding species reported in the literature to the analysis, the fauna in the research area consists of 6 subfamilies, 23 genera and 72 species. Most of the Elateridae fauna of the Western Black Sea region were classified in the subfamilies Elaterinae and Dendrometrinae. The genus *Athous* was the most species-rich genus. The species composition of the Elateridae fauna of the Western Black Sea region partially overlaps with the known Elateridae fauna of Turkey. The Western Black Sea region shares the most species with the European part of the Western Palaearctic region, including many of those in the Elateridae family, compared to other regions. Comparisons of the three geographical regions of Turkey show that fauna composition, ecological properties, and zoogeographical compositions of the Middle and Western Black Sea regions are more similar to each other than to those of the Central Anatolian region.

## Introduction

The family Elateridae is the ninth largest family of Coleoptera and belongs to the super-family Elateroidea (Lawrence 1982). The number of species of Elateridae in Turkey is increasing rapidly. According to various authors (Lawrence 1982; Booth et al. 1990; Lodos 1998; Demirsoy 1999; Laibner 2000), the family Elateridae has 6,000–10,000 described species. In Turkey, there are eight subfamilies, 65 genera, and almost 500 species belonging to Elateridae (Mertlik and Platia 2008; Kabalak and Sert 2009, 2010a, 2010b, 2011, 2012; Platia and Gudenzi 2009; Platia et al. 2009; Schimmel et al. 2009; Platia 2008, 2010a, 2010b, 2011a, 2011b, 2012; Platia et al. 2011: Sert and Kabalak 2011; Kabalak et al. 2013).

**Figure 1.**
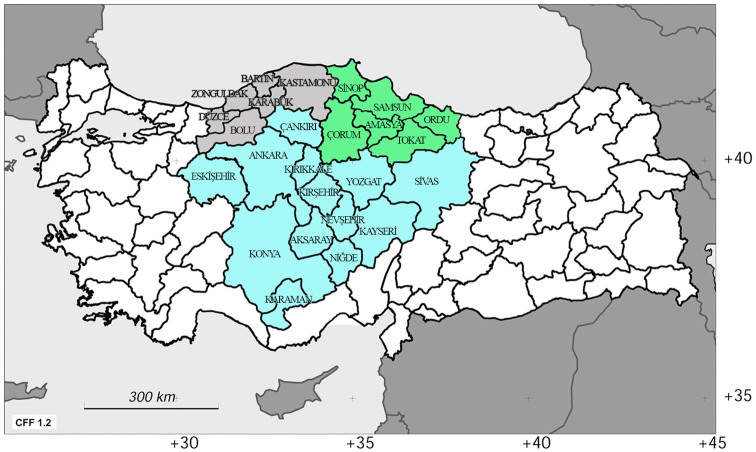
A map made in CFF (Barbier and Rasmont 1996, 2000) with modifications that shows the regions of the species collected. Grey: Western Black Sea region; light blue: Central Anatolian region; green: Middle Black Sea region. High quality figures are available online.

Turkey is at the intersection of three continents (Asia, Africa, and Europe) and three phytogeographical regions (Euro-Siberian, Irano-Turanian, and Mediterranean). It was divided into seven geographical regions (three inner regions and four coastal regions) and 21 sections of these regions at the first geography congress of Turkey in Ankara in 1941 (Atalay and Mortan 1997). The research area covered the western part of the Black Sea region, which includes the Bartin, Bolu, Düzce, Karabük, Kastamonu, and Zonguldak provinces (Figure 1). The Western Black Sea region is the part of the Black Sea region of Turkey that contains the lower part of the river basins of the Kizilirmak and Sakarya rivers, moderately high mountain ranges, and large grooves that extend between the Küre mountains, the Ilgaz, and Aladağ-Köroğlu mountain ranges. The Küre mountains, which are composed of coastal mountain ranges, extend high above the coasts. They are disrupted by the Taçköprö-Boyabat groove and the Kastamonu plateau. The Ilgaz and Aladağ-Köroğlu mountain ranges, which have summits exceeding 2500 m a.s.l., extend to the inner part of the region. The Tosya-Ilgaz-Çerkeş-Gerede-Bolu-Düzce groove, which includes the Northern Anatolian fault, extends between the Ilgaz and Aladağlar mountain ranges. Like other parts of the Black Sea region, rainfall decreases towards the inner part of the Western Black Sea region. Annual precipitation is 500 mm in the grooves, which are deprived of rainfall. Temperatures in the inner region are higher than those of the coastal regions in summer and lower than those of the coastal regions in winter. The inner region receives more snow in the winter, and less precipitation in the summer, compared to the outer region. The Western Black Sea region is covered with rotund forests, as is the Eastern Black Sea region. There are broadleaf forests, which have dense *Fagus orientalis* Lipsky and *Castanea sativa* Mill, populations in the mountainous zones. These broadleaf forests are replaced with coniferous forests (*Abies bornmülleriana* Mattf. and *Pinus sylvestris* L.) at high altitudes. Environmental conditions change suddenly towards the inner part of region, especially on the Kastamonu plateau. Oak forests are the most common at lower altitudes, and coniferous forests are common at higher altitudes on the Kastamonu plateau. *P. nigra* Arnold, and a small amount of *P. sylvestris* L. exist in the southern and southeastern mountainous parts of the region. The Bolu-Aladağlar mountains have the richest *P. sylvestris* L. forests in Turkey (Atalay and Mortan 1997; Güner et al. 2012).

The studied area is located in the Euro-Siberian and Irano-Turanian phytogeographical region. The Euro-Siberian region exists largely within the Euxine province, which extends throughout most of Caucasia and the Crimea and Dobrudja mountains. This region is essentially a belt of broadleaf deciduous forests, penetrated by conifers at higher altitudes. It is most closely related (especially in the east) to the Hyrcanian province of Northern Iran and the adjacent Talysch region, but it also has similarities to the Balkans, central Europe, and even Atlantic Europe (Davis et al. 1971). The Irano-Turanian region is by far the largest of the three regions in Turkey, and apart from a few enclaves, is confined to Central and East Anatolia. Although it is a large area rich in herbaceous and suffruticose species, it is not as well understood as the Mediterranean and Euro-Siberian regions. This is largely due to the difficulties in identifying the important genera. The broad forest zone of *Pinus nigra* Arnold, subsp. *pallasiana* (Lamb.) borders Central Anatolia in the north, west, and south. This forest meets the oak scrub (especially *Quercus pubescens* in the north and west and *Q. infectoria* Oliver sensu lato and *Q. cerris* L. in the west and south), the most abundant type of vegetation on the periphery of the central Anatolian steppes, which is usually associated with Irano-Turanian ground-flora. This Irano-Turanian scrub is most abundant in the north and west. The Irano-Turanian flora in Turkey is closely related to that of Transcaucasia, northwestern and western Iran, and northern Iraq (Davis 1965–1988).

There are some studies on the Elateridae fauna of the studied area, which were done mostly by foreign researchers. Most of these studies were limited in scope and generally consisted of descriptions of new species. Studies on Turkish Elateridae include the following: Guglielmi and Platia (1985), Platia (1989, 2003, 2004), Platia and Gudenzi (1996, 1998, 1999, 2000b, 2002, 2004, 2007), Mertlik (2000), Kabalak and Sert (2006), Mertlik and Dušánek (2006), and Mertlik and Platia (2008).

The main aim of this research was to study the faunistic composition (distributions of species, subfamilies, and genera), ecological properties (abundance, rarity, vertical distribution, habitat preference, and seasonality) of species, and the zoogeographical composition of the Elateridae fauna, which includes the zoogeographical pattern of the research area and comparisons of fauna between the Western Black Sea region and other geographical regions of Turkey.

## Materials and Methods

Elateridae specimens were collected between May and July of 2005–2009 in the Bartin, Bolu, Düzce, Karabük, Kastamonu, and Zonguldak provinces. The coordinates of the localities were recorded using GPS. The data on the localities are given in the annotated checklist below. Species' identities were determined using established keys (Gurjeva 1984; Platia 1994, 2003; Platia and Gudenzi 1998, 2000a, 2002, 2004; Laibner 2000). Specimens were deposited in the Hacettepe University Zoology Museum. In the Material Examined section, the collector's name is listed at the end as ‘col.’.

**Figure 2.**
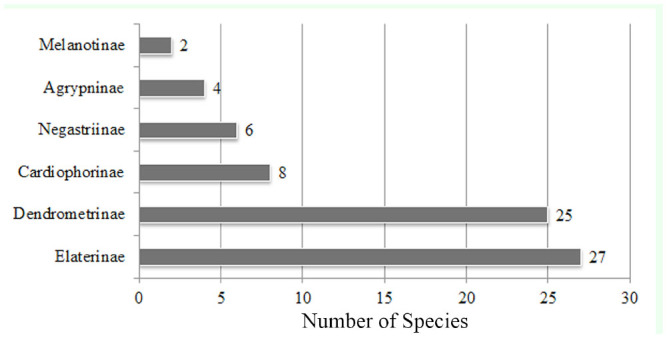
The number of species in each subfamily. High quality figures are available online.

**Figure 3.**
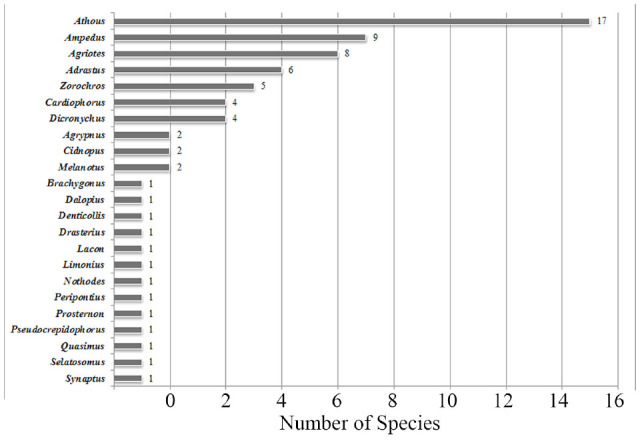
The number of species in each genus. High quality figures are available online.

**Figure 4.**
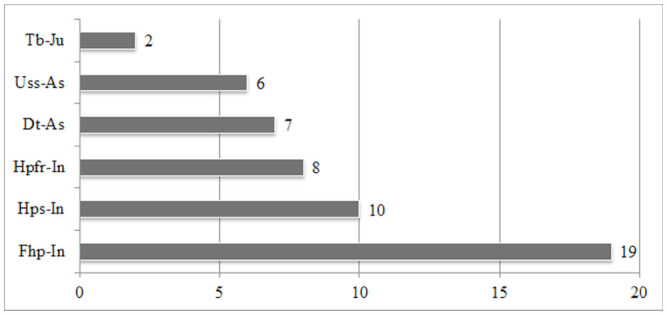
The number of species according to habitat/collection method. **Dt-As:** decaying trees by an aspirator, **Fhp-In:** forest ground herbaceous plants by an insect net, **Hpfr-In:** herbaceous plants near fields and roads by an insect net, **Hps-In:** herbacous plants near streams by an insect net, **Tb-Ju:** trees and bushes by a Japanese umbrella, and **Uss-As:** under stones near streams by an aspirator. High quality figures are available online.

**Figure 5.**
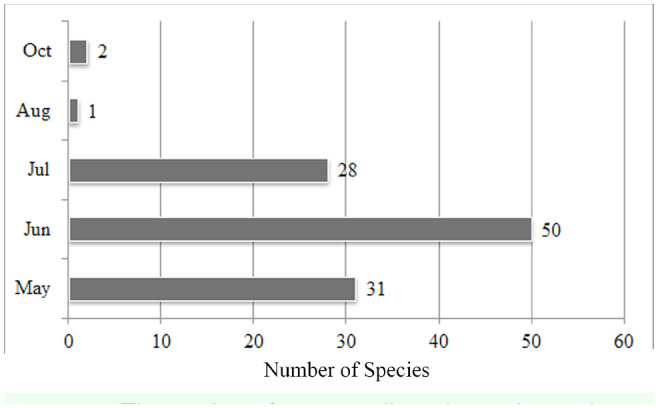
The number of species collected in each month High quality figures are available online.

**Figure 6.**
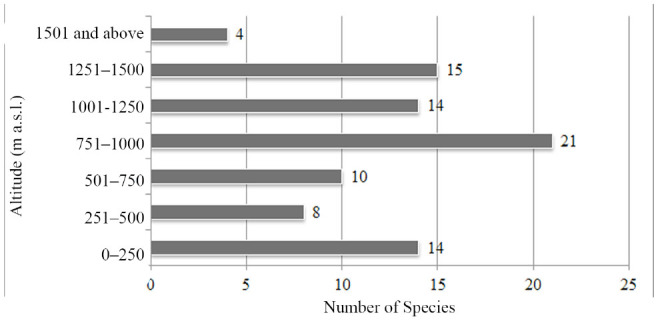
The number of species collected within each altitude zone. High quality figures are available online.

**Figure 7.**
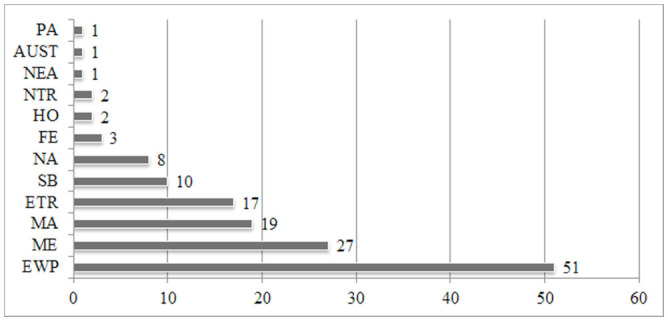
Distributions of species in various zoogeographical regions. **AUST:** Australian Region, **ETR:** Endemic species to Turkey, **EWP:** European part of the Western Palaearctic, **FE:** Far East, **HO:** Holoarctic, **MA:** Middle Asia, **ME:** Middle East, **NA:** North Africa, **NEA:** Nearctic, **NTR:** Neotropical Region, **PA:** Palaearctic, and **SB:** Siberia (Cate 2007). High quality figures are available online.

**Figure 8.**
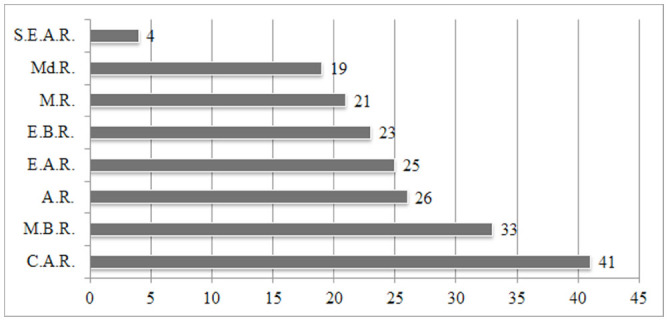
Number of species shared between the research area and other geographical regions of Turkey. **AR:** Aegean Region, **CAR:** Central Anatolian Region, **EAR:** Eastern Anatolian Region, **EBR:** Eastern Black Sea Region, **MR:** Marmara Region, **MBR:** Middle Black Sea Region, **MdR:** Mediterranean Region, **SEAR:** Southeastern Anatolian Region. High quality figures are available online.

Specimens were collected from ground herbaceous plants of the forest by an insect net (Fhp-In), herbaceous plants near streams by an insect net (Hps-In), herbaceous plants near fields and roads by an insect net (Hpfr-In), decaying trees (*Populus* spp. and *Salix* spp.) by an aspirator (Dt-As), trees and bushes by a Japanese umbrella (Tb-Ju), and under stones near streams by an aspirator (Uss-As). After finishing the fieldwork, the specimens were put in collections and the species' identities were determined. The number of specimens, the habitat/method of collection, the month of collection, the altitude of collection, the distribution in other regions of Turkey, and the zoogeographical region are given for collected species (Table 1). The province of collection, month of collection, distribution in other regions of Turkey, and zoogeographical distribution are also given for species reported in the literature (Table 2). Graphs showing the distributions of species according to their subfamily (Figure 2) and genera (Figure 3), the number of species according to the habitat/method of collection (Figure 4), the number of collected species for each month (Figure 5), the number of collected species from various altitudes (Figure 6), the zoogeographical regions of all species (Figure 7), and the number of species shared between different regions of Turkey (Figure 8) are given.

Zoogeographical region definitions were taken from Cate (2007), and the zoogeographical statuses of species in Asia were classified within four sub-regions (Middle East, Middle Asia, Siberia, and Far East). As a result, the species were sorted as those endemic to Turkey (ETr), species found in the European part of the Western Palaearctic (Ewp) region, species found in the Eastern and Western Siberian parts of Russia (Sb), Middle Eastern species (including those from Syria, Iraq, Iran, Jordan, Israel, Palestine, Lebanon, the Arabian peninsula, the Sinai peninsula, and Cyprus) (Me), Middle Asian species (including those from Afghanistan, China, Kazakhstan, Kyrgyzstan, Mongolia, Uzbekistan, Turkmenistan, and Tadzhikistan) (Ma), Far Eastern Asian (Far Eastern Territory of Russian) species (Fe), North African (Na) species, Palaearctic species (Pa), Holarctic species (Ho), Australian species (Aust), and Nearctic species (Nea) (Table 1). Zoogeographical distributions of species are as reported in Platia (1994), Penev and Alekseev (1996), Cate (2007), and Mertlik and Platia (2008). A map of the studied area was made in CFF (Barbier and Rasmont 1996, 2000). The numbers of collected males and females could not be given because of difficulties arising from separating male and female specimens.

The faunistic composition, ecological properties, and zoogeographical composition of the Western Black Sea region are compared with those from the Central Anatolian region and the Middle Black Sea region (Figure 1).

**Annotated checklist of Elateridae species of the Western Black Sea region of Turkey**
**Subfamily: Agrypninae** Candèze, 1857
**Genus: *Drasterius*** Eschscholtz, 1829
1. ***Drasterius bimaculatus*** (Rossi, 1790)
  **Examined Material: Bolu:** Seben, 41°26′12″ N, 31°35′29″ E, 857 m a.s.l., 10.VI.2009, 1 specimen; **Kastamonu:** Tosya, 41°10′16″ N, 34°03′03″ E, 1189 m a.s.l., 17. VI.2009, 1 specimen; Araç, 41°59′51″ N, 33°19′22″ E, 702 m a.s.l., 04.VI.2008, 1 specimen; **Karabük:** Ovacik, 41°02′55″ N, 32°48′22″ E, 646 m a.s.l., 16.V.2009, 3 specimens; Ovacik, 41°05′49″ N, 32°48′24″ E, 643 m a.s.l., 16.V.2009, M. Kabalak col., 2 specimens.
**Genus: *Lacon*** Castelnau de Laporte, 1838
  2. ***Lacon punctatus*** (Herbst, 1779)
  **Examined Material: Kastamonu:** Şenpazar, 41°47′16″ N, 33°10′28″ E, 566 m a.s.l., 19.VI.2009, M. Kabalak col., 1 specimen.
**Genus: *Agrypnus*** Eschscholtz, 1829
  3. ***Agrypnus crenicollis*** (Ménétriés, 1832)
**Examined Material: Kastamonu:** Tosya, 41°10′16″ N, 34°03′03″ E, 1189 m a.s.l., 17.VI.2009, M. Kabalak col., 1 specimen.
**Subfamily: Dendrometrinae** Gistel, 1856
**Genus: *Limonius*** Eschscholtz, 1829
  4. ***Limonius minutus* (**Linnaeus, 1758)
  **Examined Material: Bolu:** Gerede, 40°48′25″ N, 32°11′18″ E, 1400 m a.s.l., 07.VI.2009, 5 specimens; Gerede, 40°48′51″ N, 32°12′00″ E, 1674 m a.s.l., 07.VI.2009, 1 specimen; Gerede, 40°48′55″ N, 32°11′56″ E, 1656 m a.s.l., 07.VI.2009, 1 specimen; **Kastamonu:** Araç, 41°18′43″ N, 33°35′53″ E, 1132 m a.s.l., 18.VI.2009, 1 specimen; **Zonguldak:** Saltukova, 10.VI.1994, M. Kabalak col., 1 specimen.
**Genus: *Nothodes*** LeConte, 1861
  5. ***Nothodes parvulus*** (Panzer, 1799)
  **Examined Material: Karabük:** Safranbolu, 41°18′35″ N, 32°40′59″ E, 782 m a.s.l., 17.V.2009, M. Kabalak col., 1 specimen.
**Genus: *Athous*** Eschscholtz, 1829
  **Subgenus: *Athous*** Eschscholtz, 1829
  6. ***Athous* (*A*.) *haemorrhoidalis*** (Fabricius, 1801)
  **Examined Material: Karabük:** Eskipazar, 40°51′56″ N, 32°38′35″ E, 985 m a.s.l., 16.V.2009, M. Kabalak col., 1 specimen.
**Subgenus: *Haplathous*** Reitter, 1905
  7. ***Athous (H.) marginicollis*** Reitter, 1890
  **Examined Material: Karabük:** Eskipazar, 40°57′14″ N, 32°25′23″ E, 1263 m a.s.l., 07.VI.2009, M. Kabalak col., 1 specimen.
8. ***Athous (H.) subfuscus*** (O. F. Müller, 1764)
  **Examined Material: Bolu:** Central County, 40°55′53″ N, 31°43′08″ E, 1370 m a.s.l., 08.VI.2009, 1 specimen; Gerede, 40°48′25″ N, 32°11′18″ E, 1400 m a.s.l., 07.VI.2009, M. Kabalak col., 7 specimens.
**Subgenus: *Orthathous*** Reitter, 1905
  9. ***Athous (O.) daccordii*** Guglielmi and Platia, 1985
  **Examined Material: Kastamonu:** Azdavay, 41°41′16″ N, 33°23′59″ E, 906 m a.s.l., 18.VI.2009, M. Kabalak col., 1 specimen.
10. ***Athous (O.) paflagonensis*** Platia and Gudenzi, 1998
  **Examined Material: Bartin:** Central County, 41°41′59″ N, 32°40′26″ E, 375 m a.s.l., 14.VII.2008, 8 specimens; **Karabük:** Safranbolu, 41°28′18″ N, 32°40′23″ E, 271 m a.s.l., 13.VII.2008, M. Kabalak col., 2 specimens.
  11. ***Athous (O.) warchalowskii*** Platia and Tarnawski, 1998
  **Examined Material: Bartin:** Central County, 41°39′59″ N, 32°13′29″ E, 7 m a.s.l., 18.V.2009, 2 specimens; Ulus road Küllü village, 12.VI.2006, 1 specimen; Central County, 41°27′35″ N, 32°15′32″ E, 76 m a.s.l., 10.VI.2006, 3 specimens; **Bolu:** Central County, 40°50′53″ N, 31°40′12″ E, 1403 m a.s.l., 22.VII.2008, 11 specimens; **Karabük:** Safranbolu, 41°28′18″ N, 32°40′23″ E, 271 m a.s.l., 13.VII.2008, M. Kabalak col., 1 specimen.
**Genus: *Denticollis*** Piller and Mitterpacher, 1783
  12. ***Denticollis parallelicollis*** Aubé, 1850
  **Examined Material: Bolu:** Yedigöller Natural Park, 40°57′16″ N, 31°44′47″ E, 1360 m a.s.l., 08.VI.2009, M. Kabalak col., 1 specimen.
**Genus: *Prosternon*** Latreille, 1834
  13. ***Prosternon tessellatum*** (Linnaeus, 1758)
  **Examined Material: Karabük:** Eskipazar, 40°57′14″ N, 32°25′23″ E, 1263 m a.s.l., 07.VI.2009, 1 specimen; **Kastamonu:** Central County, 41°11′09″ N, 34°00′06″ E, 1263 m a.s.l., 17.VI.2009, 1 specimen; Central County, 41°22′08″ N, 33°45′20″ E, 894 m a.s.l., 18.VI.2009, M. Kabalak col., 1 specimen
**Genus: *Pseudocrepidophorus*** Dolin and Agajev, 1988
  ***Pseudocrepidophorus flavescens*** (Eschscholtz, 1818) **Examined Material: Kastamonu:** Inegöl, 41°59′13″ N, 33°33′14″ E, 635 m a.s.l., 19.VI.2009 M. Kabalak col., 1 specimen.
**Subfamily: Elaterinae** Leach, 1815
**Genus: *Ampedus*** Dejean, 1833
  14. ***Ampedus anatolicus*** Platia and Gudenzi, 2002
  **Examined Material: Kastamonu:** Tosya, 40°58′49″ N, 34°11′06″ E, 1147 m a.s.l., 18.V.2008, M. Kabalak col., 2 specimens.
  15. ***Ampedus cinnaberinus*** (Eschscholtz, 1829)
  **Examined Material: Bolu:** Kibrisçik, 40°25′14″ N, 31°50′49″ E, 924 m a.s.l., 02.V.2009, 1 specimen; **Kastamonu:** Tosya, 40°58′49″ N, 34°11′06″ E, 1147 m a.s.l., 18.V.2008, M. Kabalak col., 2 specimens.
  16. ***Ampedus elegantulus*** (Schönherr, 1817)
  **Examined Material: Bartin:** Kozcağiz, 41°24′45″ N, 32°20′28″ E, 162 m a.s.l., 17.V.2009, M. Kabalak col., 1 specimen.
  17. ***Ampedus platiai*** Schimmel, 1990
  **Examined Material: Karabük:** Eskipazar, 40°54′38″ N, 32°42′33″ E, 1154 m a.s.l., 16.V.2009, M. Kabalak col., 2 specimens.
  18. ***Ampedus pomorum*** (Herbst in Füssly, 1784)
  **Examined Material: Karabük:** Eflani, 41°25′16″ N, 32°48′52″ E, 938 m a.s.l., 17.V.2009, M. Kabalak col., 1 specimen.
  19. ***Ampedus (A.) praeustus*** (Fabricius, 1792)
  **Examined Material: Kastamonu:** Tosya, 40°57′25″ N, 34°12′32″ E, 1489 m a.s.l., 20.V.2007, M. Kabalak col., 14 specimens.
  20. ***Ampedus (A.) rufipennis*** (Stephens, 1830)
  **Examined Material: Bolu:** Merkez, 40°39′34″ N, 31°24′55″ E, 868 m a.s.l., 03.V.2009, M. Kabalak col., 1 specimen.
**Genus: *Synaptus*** Eschscholtz, 1829
  21. ***Synaptus filiformis* (**Fabricius, 1781)
  **Examined Material: Bartin:** Central County, 41°38′15″ N, 32°19′53″ E, 10 m a.s.l., 10.VI.2006, 1 specimen; **Bolu:** Central County, 40°47′35″ N, 31°38′29″ E, 916 m a.s.l., 08.VI.2009, 2 specimens; **Karabük:** Eskipazar, 40°51′29″ N, 32°38′01″ E, 1295 m a.s.l., 16.V.2009, M. Kabalak col., 1 specimen.
**Genus: *Peripontius*** Gurjeva, 1979
  22. ***Peripontius terminatus*** (Erichson, 1842)
  **Examined Material: Bartin:** Ulus, 41°35′50″ N, 32°41′53″ E, 213 m a.s.l., 13.VII.2008, M. Kabalak col., 1 specimen.
**Genus: *Adrastus*** Eschscholtz, 1829
  23. ***Adrastus anatolicus*** Platia and Schimmel, 1991
  **Examined Material: Bartin:** Central County, 41°38′15″ N, 32°19′53″ E, 10 m a.s.l., 10.VI.2006, 14 specimens; **Bolu:** Göynük, 40°27′07″ N, 30°55′45″ E, 894 m a.s.l., 21.VII.2008, M. Kabalak col., 2 specimens.
24. ***Adrastus circassicus*** Reitter, 1896
  **Examined Material: Kastamonu:** Şenpazar, 41°47′16″ N, 33°10′28″ E, 566 m a.s.l.,1 9.VI.2009, 1 specimen; Cide, 41°51′57″ N, 33°03′13″ E, 634 m a.s.l., 19.VI.2009, 15 specimens; Doğanyurt, 41°59′13″ N, 33°33′14″" E, 635 m a.s.l., 19.VI.2009, M. Kabalak col., 1 specimen.
  25. ***Adrastus montanus*** (Scopoli, 1763)
  **Examined Material: Bartin:** Central County, 41°27′35″ N, 32°15′32″ E, 76 m a.s.l., 10.VI.2006, M. Kabalak col., 1 specimen.
**Genus: *Agriotes*** Eschscholtz, 1829
  27. ***Agriotes acuminatus*** (Stephens, 1830)
  **Examined Material: Bolu:** Central County, 40°54′20″ N, 31°40′39″ E, 1539 m a.s.l., 08.VI.2009, 1 specimen; **Düzce:** Yiğilca, 40°59′02″ N, 31°39′42″ E, 1185 m a.s.l., 19.VI.2009, 3 specimens; Kaynaşli, 40°40′07″ N, 31°16′17″ E, 920 m a.s.l., 09.VI.2009, M. Kabalak col., 1 specimen.
  28. ***Agriotes infuscatus*** Desbrochers des Loges, 1870
  **Examined Material: Bartin:** Kumluca, 41°23′50″ N, 32°29′04″ E, 511 m a.s.l., 18.V.2009, 1 specimen; **Bolu:** Central County, 40°56′17″ N, 31°44′44″ E, 1366 m a.s.l., 08.VI.2009, 3 specimens; Central County 40°39′34″ N, 31°24′55″ E, 868 m a.s.l., 03.V.2009, 5 specimens; Central County, 40°51′35″ N, 31°41′00″ E, 1353 m a.s.l., 08.VI.2009, 1 specimen; Central County, 40°55′53″ N, 31°43′08″ E, 1370 m a.s.l., 08.VI.2009, 4 specimens; Central County, 40°47′35″ N, 31°38′29″ E, 916 m a.s.l., 08.VI.2009, 1 specimen; **Düzce:** Yiğilca, 40°59′02″ N, 31°39′42″ E, 1185 m (9 ex.) 08.VI.2009, Kaynaşli (40°39′45″ N 31°16′14″ E) 1050 m a.s.l., 09.VI.2009, 3 specimens; Kaynaşli, 40°40′07″ N, 31°16′17″ E, 920 m a.s.l., 09.VI.2009, 5 specimens; Gölyaka, 40°43′08″ N, 31°02′47″ E, 720 m a.s.l., 09.VI.2009, 3 specimens; **Karabük:** Safranbolu, 41°21′58″ N, 32°46′03″ E, 940 m a.s.l., 17.V.2009, 2 specimens; Safranbolu, 41°18′35″ N, 32°40′59″ E, 782 m a.s.l., 17.V.2009, 1 specimen; **Kastamonu:** Central County, 41°20′09″ N, 33°38′14″ E, 1221 m a.s.l., 18.VI.2009, M. Kabalak col., 1 specimen.
  29. ***Agriotes paludum*** Kiesenwetter, 1859
  **Examined Material: Bartin:** Kozcağiz, 41°19′15″ N, 32°22′52″ E, 462 m a.s.l., 17.VII.2008, 2 specimens; Ulus, 41°28′40″ N, 32°33′09″ E, 483 m a.s.l., 13.VII.2008, 1 specimen; Central County, 41°27′35″ N, 32°15′32″ E), 76 m a.s.l., 10.VI.2006, 1 specimen; **Karabük:** Safranbolu, 41°18′35″ N, 32°40′59″ E, 782 m a.s.l., 17.V.2009, M. Kabalak col., 1 specimen.
  30. ***Agriotes proximus*** Schwarz, 1891
  **Examined Material: Bartin:** Central County, 41°37′59″ N, 32°11′43″ E, 371 m a.s.l., 18.V.2009, 1 specimen; Central County, 41°27′35″ N, 32°15′32″ E, 76 m a.s.l., 10.VI.2006, 2 specimens; Kozcağiz, 41°19′15″ N, 32°22′52″ E, 462 m a.s.l., 17.VII.2009, 2 specimens; Ulus, 41°28′40″ N, 32°33′09″ E, 483 m 13.VII.2008, 1 specimen; **Bolu:** Central County, 40°38′19″ N, 31°48′29″ E, 1516 m a.s.l., 03.V.2009, 6 specimens; **Karabük:** Eskipazar, 40°54′38″ N, 32°42′33″ E, 1154 m a.s.l., 16.V.2009, 1 specimen; Eskipazar, 40°51′29″ N, 32°38′01″ E, 1295 m a.s.l., 16.V.2009, 1 specimen; Safranbolu,
  41°21′58″ N, 32°46′03″ E, 940 m a.s.l., 17.V.2009, 1 specimen; Eskipazar, 40°51′56″ N, 32°38′35″ E, 985 m a.s.l., 16.V.2009, 1 specimen; Eskipazar, 40°56′30″ N, 32°31′21″ E, 730 m a.s.l., 07.VI.2009, 1 specimen; **Düzce:** Kaynaş, 40°39′45″ N, 31°16′14″ E, 1050 m a.s.l., 09.VI.2009, 1 specimen; Cumayeri, 40°49′45″ N, 30°59′23″ E, 169 m a.s.l., 09.VI.2009, 1 specimen; Yiğilca 40°59′02″ N, 31°39′42″ E, 1185 m a.s.l., 08.VI.2009, M. Kabalak col., 1 specimen.
  31***. Agriotes sputator*** (Linnaeus, 1758)
  **Examined Material: Bartin:** Central County, 41°19′15″ N, 32°22′52″ E, 462 m a.s.l., 10.VI.2006, 1 specimen; Ulus, 41°34′40″ N, 32°36′58″ E, 298 m a.s.l., 10.VI.2006, 1 specimen; Ulus, 41°33′05″ N, 32°36′28″ E, 159 m a.s.l., 12.VI.2006, 1 specimen; Ulus, 41°39′29″ N, 32°44′39″ E, 301 m a.s.l., 13.VII.2008, 1 specimen; Kozcağiz, 41°24′45″ N, 32°20′28″ E, 162 m a.s.l., 17.V.2009, 1 specimen; **Düzce:** Kaynaşh, 40°40′07″ N, 31°16′17″ E, 920 m a.s.l., 09.VI.2009, 1 specimen; **Karabük:** Eskipazar, 40°51′56″ N, 32°38′35″ E, 985 m a.s.l., 16.V.2009, 1 specimen; Eskipazar, 40°57′14″ N, 32°25′23″ E, 1263 m a.s.l., 07.VI.2009, 1 specimen; **Kastamonu:** Central County, 41°15′29″ N, 33°51′44″ E, 1022 m a.s.l., 17.VI.2009, M. Kabalak col., 2 specimens.
32. ***Agriotes ustulatus*** (Schaller, 1783)
  **Examined Material: Kastamonu:** Cide, 41°51′57″ N, 33°03′13″ E, 634 m a.s.l., 19.VI.2009, M. Kabalak col., 2 specimens.
  **Genus: *Dalopius*** Eschscholtz, 1829
  33. ***Dalopius marginatus*** (Linnaeus, 1758)
  **Examined Material: Bolu:** Central County, 40°51′35′ N, 31°41′00″ E, 1353 m a.s.l., 08.VI.2009, 2 specimens; **Düzce:** Kaynaşli, 40°40′07″ N, 31°16′17″ E), 920 m a.s.l., 09.VI.2009, 1 specimen; **Karabük:** Eskipazar, 40°57′14″ N, 32°25′23″ E, 1263 m a.s.l., 07.VI.2009, M. Kabalak col., 1 specimen.
**Subfamily: Negastriinae** Nakane and Kishii 1956
**Genus: *Quasimus*** Gozis, 1886
  34. ***Quasimus minutissimus*** (Germar, 1817)
  **Examined Material: Bartin:** Central County, 41°39′56″ N, 32°13′48″ E, 167 m a.s.l., 22.V.2005, 41 specimens; Kozcağiz, 41°24′45″ N, 32°20′28″ E, 162 m a.s.l., 17.V.2009, 10 specimens; Central County, 41°37′59″ N, 32°11′43″ E, 371 m a.s.l., 18.V.2009, 10 specimens; Central County, 41°37′12″ N, 32°10′06″ E, 161 m a.s.l., 18.V.2009, 4 specimens; Central County, 41°36′44″ N, 32°09′45″ E, 50 m a.s.l., 18.V.2009, 10 specimens; Central County, 41°39′23″ N, 32°13′35″ E, 205 m a.s.l., 12.VI.2006, 2 specimens; Central County, 41°39′54″ N, 32°13′45″ E, 160 m a.s.l., 22.V.2005, 38 specimens; Central County, 41°39′16″ N, 32°13′25″ E, 22.V.2005, 214 m a.s.l., 4 specimens; Central County, 41°36′53″ N, 32°26′26″ E, 34 m a.s.l., 22.V.2005, 2 specimens; Central County 41°39′61″ N, 32°13′07″ E, 17 m a.s.l., 22.V.2005, 1 specimen; Central County, 41°39′57″ N, 32°13′28″ E, 14 m a.s.l., 22.V., 1 specimen; **Karabük:** Eflani, 41°25′21″ N, 32°48′37″ E, 915 m a.s.l., 17.V.2009, 17 specimens; Safranbolu, 41°26′05″ N, 32°46′04″ E, 428 m a.s.l., 17.V.2009, 13 specimens; Eskipazar, 40°57′14″ N, 32°25′23″ E, 1263 m a.s.l., 07.VI.2009, 1 specimen; **Kastamonu:** Cide, 41°51′57″ N, 33°03′13″ E, 634 m a.s.l., 19.VI.2009, 66 specimens; Araç, 41°21′04″ N, 33°20′25″ E, 1115 m a.s.l., 18.VI.2009, 9 specimens; İnebolu, 41°49′53″ N, 33°42′33″ E, 646 m a.s.l., 19.VI.2009, 3 specimens; Central County, 41°11′09″ N, 34°00′06″ E, 1263 m a.s.l., 17. VI.2009, 53 specimens; Central County, 41°22′08″ N, 33°45′20″ E, 894 m a.s.l., 18.VI.2009, 1 specimen , Araç, 41°21′04″ N, 33°20′25″ E, 1115 m a.s.l., 18.VI.2009, M. Kabalak col., 9 specimens.
**Genus: *Zorochros*** C. G. Thomson, 1859
  35. ***Zorochros alysidotus*** (Kiesenwetter, 1858)
  **Examined Material: Karabük:** Yenice, 41°14′01″ N, 32°21′39″ E, 197 m a.s.l., 17.V.2009, M. Kabalak col., 17 specimens.
  36. ***Zorochros meridionalis*** (Laporte de Castelnau, 1840)
  **Examined Material: Düzce:** Akçakoca, 41°01′04″ N, 30°59′58″ E, 45 m a.s.l., 09.VI.2009, 1 specimen; **Karabük:** Ovacik, 41°05′49″ N, 32°48′24″ E, 643 m a.s.l., 16.V.2009, 12 specimens; Yenice, 41°18′39″ N, 32°22′45″ E, 331 m a.s.l., 17.V.2009, 15 specimens; Yenice, 41°14′01″ N, 32°21′39″ E, 197 m a.s.l., 17.V.2009, 1 specimen; **Kastamonu:** Araç, 41°59′51″ N, 33°19′22″ E, 702 m a.s.l., 04.VI.2008, 4 specimens; Araç, 41°02′14″ N, 33°18′51″ E, 702 m a.s.l., 04.VI.2008, 1 specimen; Tosya, 41°10′16″ N, 34°03′03″ E, 1189 m a.s.l., 17.VI.2009, M. Kabalak col., 5 specimens.
  37. ***Zorochros pilosellus*** (Reitter, 1895)
  **Examined Material: Kastamonu:** Araç, 40°59′51″ N, 33°19′22″ E, 702 m a.s.l., 04.VI.2008 4 specimens; Araç, 41°02′14″ N, 33°18′51″ E, 702 m a.s.l., 04.VI.2008, M. Kabalak col., 12 specimens.
  38. ***Zorochros stibicki*** (Leseigneur, 1970)
  **Examined Material: Düzce:** Akçakoca, 41°01′04″ N, 30°59′58″ E, 45 m a.s.l., 09.VI.2009, 7 specimens; **Karabük:** Eskipazar, 40°51′29″ N, 32°38′01″ E, 1295 m a.s.l., 16.V.2009, M. Kabalak col., 1 specimen.
**Subfamily: Cardiophorinae** Candèze, 1860
**Genus: *Cardiophorus*** Eschscholtz, 1829
  **Subgenus: *Cardiophorus*** Eschscholtz, 1829
  39. ***Cardiophorus*** (C.) ***anticus*** Erichson, 1840
  **Examined Material: Karabük:** Eflani, 41°25′16″ N, 32°48′52″ E, 938 m a.s.l., 17.V.2009, M. Kabalak col., 1 specimen.
  40. ***Cardiophorus dolini*** Mardjanian, 1985
  **Examined Material: Bolu:** Seben, 40°26′12″ N, 31°35′29″ E, 857 m a.s.l., 10.VI.2009, 1 specimen; **Karabük:** Safranbolu, 41°28′18″ N, 32°40′23″ E, 271 m a.s.l., 13.VII.08, M. Kabalak col., 1 specimen.
  41. ***Cardiophorus (C.) vestigialis*** Erichson, 1840
  **Examined Material: Kastamonu:** Merkez, 41°20′09″ N, 33°38′14″ E, 1221 m a.s.l., 18.VI.2009, M. Kabalak col., 1 specimen.
  **Genus: *Dicronychus*** Brullè, 1832
  42. ***Dicronychus cinereus*** (Herbst, 1784)
  **Examined Material: Bolu:** Central County, 40°47′35″ N, 31°38′29″ E, 916 m a.s.l., 08.VI.2009, M. Kabalak col., 1 specimen.
  43. ***Dicronychus obscuripennis*** (Pic, 1899)
  **Examined Material: Bartin:** Central County, 41°40′25″ N, 32°13′49″ E, 16 m a.s.l., 21.V.2005, 1 specimen; **Kastamonu:** Tosya, 40°58′49″ N, 34°ll′06″ E, 1147 m a.s.l., 18.V.2008, M. Kabalak col., 1 specimen.
  44. ***Dicronychus senaci*** Desbrochers des Loges, 1870
  **Examined Material: Bolu:** Gerede, 40°48′51″ N, 32°12′00″ E, 1674 m a.s.l., 07.VI.09, 16 specimens; Merkez, 40°47′35″ N, 31°38′29″ E, 916 m a.s.l., 08.VI.09, 2 specimens; **Kastamonu:** Merkez, 41°11′09″ N, 34°00′06″ E, 1263 m a.s.l., 17.VI.2009, M. Kabalak col., 2 specimens.

## Results and Discussion

### Faunistic composition of the Western Black Sea region

Species of the subfamilies Elaterinae (27 species, 37.5%) and Dendrometrinae (25 species, 34.7%) comprise most of the studied area's Elateridae fauna. The numbers of species in the subfamilies Cardiophorinae, Negastriinae, Agrypninae, and Melanotinae are shown in Figure 2. The number of species in the genera are shown in Figure 3.

Distributions of species, according to their genera, are compared with the Elateridae fauna of Turkey in Table 3. The distributions of species in each genus show that the Elateridae fauna found in this study partially overlap with the Elateridae fauna of Turkey.

### Ecological properties of the Elateridae fauna of the Western Black Sea region of Turkey

In total, 614 specimens were collected. ***Quasimus minutissimus*** (295 specimens) was the most abundant species. ***Adrastus montanus, Agrypnus crenicollis, Ampedus (Ampedus) elegantulus, A. (A.) pomorum, A, (A.) rufipennis, Athous (Athous) haemorrhoidalis, A. (Haplathous) marginicollis, A. (Orthathous) daccordii, Cardiophorus (Cardiophorus) anticus, C. (C.) vestigialis, Denticollis parallelicollis, Dicronychus cinereus, Lacon punctatus, Nothodes parvulus, Peripontius terminatus,*** and ***Pseudocrepidophorus flavescens*** were the rarest species, which were represented with one specimen each. More frequently collected species may have dense populations, and less frequently collected species may have sparse populations in nature. On the other hand, there are three additional possibilities for these results. Species may have been collected on dates with abnormally low or high density populations, the habitat chosen for collection may have had an abnormal population density, and certain species may have been collected more or less frequently by particular collecting methods.

Variable numbers of species were collected from various habitats using different collecting methods (Table 1 and Figure 4). More than half of the species were collected from forest habitats. This result parallels that of the wealth of the Western Black Sea forest area and draws attention to the importance of protecting the forests in the studied area. Using additional collection methods (light trap, window trap, pitfall trap, etc.) in future studies may yield information about habitat preferences and the activity periods (nocturnal species, diurnal species, etc.) of Elateridae species in the research area. Species distributions by collection month are given in Table 1 and Figure 5.

Species were collected from various altitudes. An evaluation of the vertical distributions of species was made in terms of seven vertical 250 m intervals (A to G) from 0 to 1674 m a.s.l. as shown in Table 1. The results showed differences in the vertical distributions of species. In addition, the collection frequencies of species from each interval were different. The most diverse range was the D interval, with 21 species, and it was followed by the F interval (15 species), the A and E intervals (14 species each), the C interval (10 species), the B interval (8 species), and the G interval (4 species) (Figure 6). *Agriotes proximus*, which was the most widely distributed species, was present in all intervals. *Quasimus minutissimus*, which was the second most widely distributed species, was present in all vertical intervals except G.

### Zoogeographical composition of the Elateridae fauna of the Western Black Sea region of Turkey

According to the literature, 17 species are endemic to Turkey. The rest of the species are also distributed in the European part of the Western Palaearctic (51 species), the Middle East (27 species), Middle Asia (19 species), Siberia (10 species), North Africa (8 species), the Far East (3 species), the Neotropic (2 species), the Holarctic (2 species), the Palaearctic (1 species), the Nearctic (1 species), and Australia (1 species). Therefore, the fauna of Turkey have important relationships with the fauna of Europe, North Africa, and Asia. The studied area shares the most species (51 species) with the European part of the Western Palaearctic, which is probably due to their similar floristic and climatic features. Asia (the Far East, Middle Asia, the Middle East, and Siberia) comes after the European part of the Western Palaearctic with 35 species shared with Turkey. According to Penev and Alekseev (1996) and Cate (2007), some recorded species are distributed in the Australian, Nearctic, Neotropic, Palaearctic (Cardiophorus ***vestigialis),*** and Holarctic (***Agriotes sputator*** and ***Agrypnus murinus)*** regions as well as the whole Palaearctic region except for North Africa (***Agriotes lineatus).*** According to Cate (2007), comparisons between the fauna of the studied area and those of the Western Palaearctic countries and territories show that the studied area shares the most species with Greece (47 species) and Italy (42 species). It also shares species with Bulgaria and the Southern European territory of Russia (41 species each), France (38 species), Hungary and Romania (37 species each), Austria, Croatia, Germany, and Spain (35 species each), the Czech Republic, Slovakia, and Slovenia (34 species each), Switzerland and Ukraine (33 species each), Azerbaijan, Armenia, Georgia, and Poland (31 species each), and Belgium and Moldavia (29 species each). On the other hand, despite its close proximity, the studied area shares few species with Iran (23 species), Syria (13 species), and Iraq (1 species). This may be explained by the floristic, faunistic, and climatic similarities between European countries and the Western Black Sea area.

Distributions of species in other regions of Turkey are reported in Tables 1 and 2. According to the literature, the studied area shares 41 species with the Central Anatolian region, 33 species with the Middle Black Sea region, 26 species with the Aegean region, 25 species with the Eastern Anatolian region, 23 species with the Eastern Black Sea region, 21 species with the Marmara region, 19 species with the Mediterranean region, and 4 species with the South Eastern Anatolian region of Turkey (Figure 8). These results indicate that the studied area shares most of its species with the Central Anatolian region, likely due to its geographical proximity.

### Comparisons with the Central Anatolian and the Middle Black Sea regions

The results of the faunistic composition, ecological properties, and zoogeographical composition of the Western Black Sea region were compared with previous studies conducted in the Central Anatolian region (Kabalak and Sert 2011) and the Middle Black Sea region (Sert and Kabalak 2011). According to comparisons of faunistic compositions, the richest subfamily in all three of these regions is the subfamily Elaterinae. The second and third richest subfamilies are the same for the Western and Middle Black Sea regions (Dendrometrinae and Cardiophorinae) but in the opposite order for the Central Anatolian region (Cardiophorinae and Dendrometrinae). Species richness in the three regions is similar. The genera ***Cardiophorus, Agriotes,*** and ***Ampedus*** are abundant in the Central Anatolian region, while the genera ***Athous, Ampedus,*** and ***Agriotes*** are abundant in the Western and Middle Black Sea regions. ***Athous*** is the most abundant genus in the Western Black Sea region.

According to comparisons of ecological properties, the most abundant species in the Central Anatolian region is ***Agriotes paludum,*** while it is ***Quasimus minutissimus*** in the Middle and Western Black Sea regions. In the Central Anatolian region, the highest number of the species was collected from herbaceous plants near streams (Hps), whereas most species were collected from ground herbaceous plants of forests (Fhp) in both the Middle and Western Black Sea regions. The highest numbers of species were recorded in May in the Central Anatolian region and the Middle Black Sea region, whereas the highest number of species was recorded in June in the Western Black Sea region. In the Central Anatolian and Middle Black Sea regions, the highest number of species was collected from interval E (1001–1250 m a.s.l.), whereas the highest number of species was collected from interval D (751–1000 m a.s.l.) in the Western Black Sea region. There were differences in the vertical distributions of species in the Central Anatolian (***Agriotes paludum),*** Middle Black Sea (***Adrastus anatolicus),*** and Western Black Sea (***Agriotes proximus)*** regions.

Zoogeographical compositions of the Central Anatolian, Middle Black Sea, and Western Black Sea regions are very similar. These regions, followed by the Middle East and Middle Asia, share the most species with the European part of the Western Palaearctic region. On the other hand, the Western Black Sea region has the highest number of endemic species among these three regions. There are some additional differences between these regions. The Central Anatolian region shares the highest number of species with the Mediterranean region, whereas the Middle and Western Black Sea regions share the highest number of species with the Central Anatolian region. This may seem contradictory; however, this could be explained by the different faunistic structures of these regions. Comparisons of the three regions show that the faunistic structures, ecological properties, and zoogeographical compositions of the Middle and Western Black Sea regions are more similar to each other than to those of the Central Anatolian region. Similar geographical, climatic, and floristic properties of the Middle and Western Black Sea regions are likely the main reasons for the faunistic, ecological and zoogeographical similarities. Turkey has rich Elateridae fauna, and many new species were reported recently. In addition, there is a lack of comprehensive studies in different geographical regions of Turkey. Future studies, including descriptions of new species of other geographical regions of Turkey, can extend the results of this research.

## 

**Table 1.**
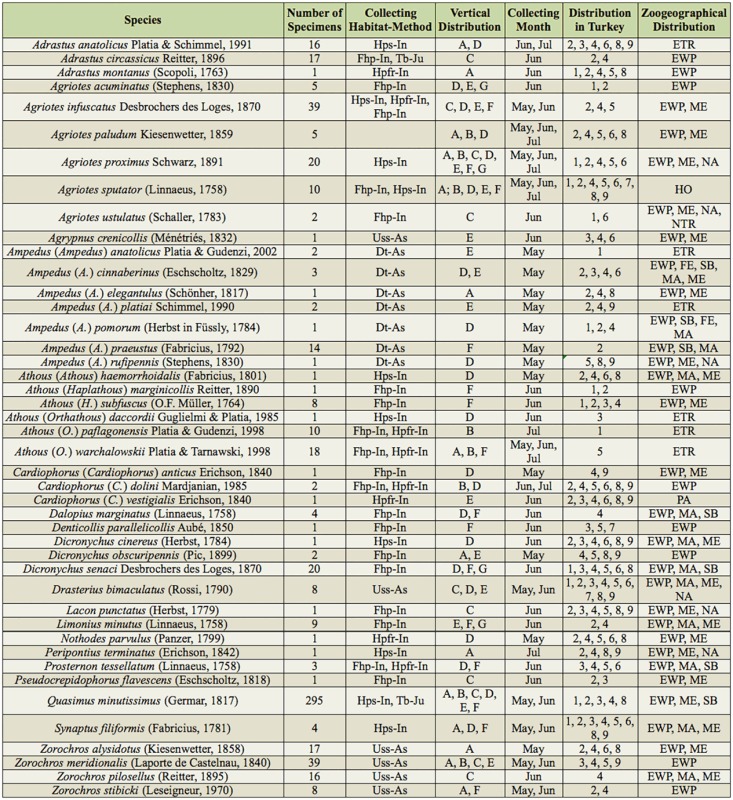
Number of specimens, the collection/habitat method, and the distributions of species. **Collecting habitat-method: Fhp-In:** Forest ground herbaceous plants-Insect net, **Hps-In:** Herbaceous plants near streams-Insect net, **Hpfr-In:** Herbaceous plants near fields and roads-Insect net, **Tb-Ju:** Trees and bushes-Japenese Umbrella, **Dt-As:** Decaying trees-Aspirator, **Uss-As:** Under stones and detritus near stream-Aspirator, **Usp-As:** Under stones and plants-Aspirator. **Vertical distribution** (m a.s.l.): **A:** 0–250m, **B:** 251–500, **C:** 501–750, **D:** 751–1000, **E:** 1001–1250, **F:** 1251–1500, **G:** 1501–1750m. **Collecting months: May:** May, **Jun:** June, **Jul:** July, **Aug:** August, **Oct:** October. **Distributions in Turkey: I:** Western Black Sea Region, **2:** Middle Black Sea Region, **3:** Eastern Black Sea Region, **4:** Central Anatolian Region, **5:** Marmara Region, **6:** Eastern Anatolian Region, **7:** South Eastern Anatolian Region, **8:** Aegean Region, **9:** Mediterranean Region (Dušánek and Mertlik 2007; Guglielmi and Platia 1985; Gülperçin and Tezcan 2009 and 2010; Kabalak 2010; Kabalak and Sert 2005, 2006, 201la, 201lb; Kesdek et al. 2006; Lohse 1979; Mertlik 2000; Mertlik and Dušánek 2006; Mertlik and Platia 2008; Platia 1989, 2003; Platia and Gudenzi 1996, 1998, 2000a, 2000b, 2002, 2004; Platia and Schimmel 1991; Schimmel 1990; Sert and Kabalak 2011; Yüksel 1970); **Zoogeographical Distibutions: AUST:** Australian, **ETR:** Endemic for Turkey, **EWP:** European part of western Palaearctic, **FE:** Far East, **HO:** Holarctic, **MA:** Middle Asia, **ME:** Middle East, **NA:** North Africa, **NEA:** Nearctic, **NTR:** Neotropic, **PA:** Palearctic, **SB:** Siberia (Cate 2007; Mertlik and Platia 2008; Penev and Alekseev 1996; Platia 1994).

**Table 2.**
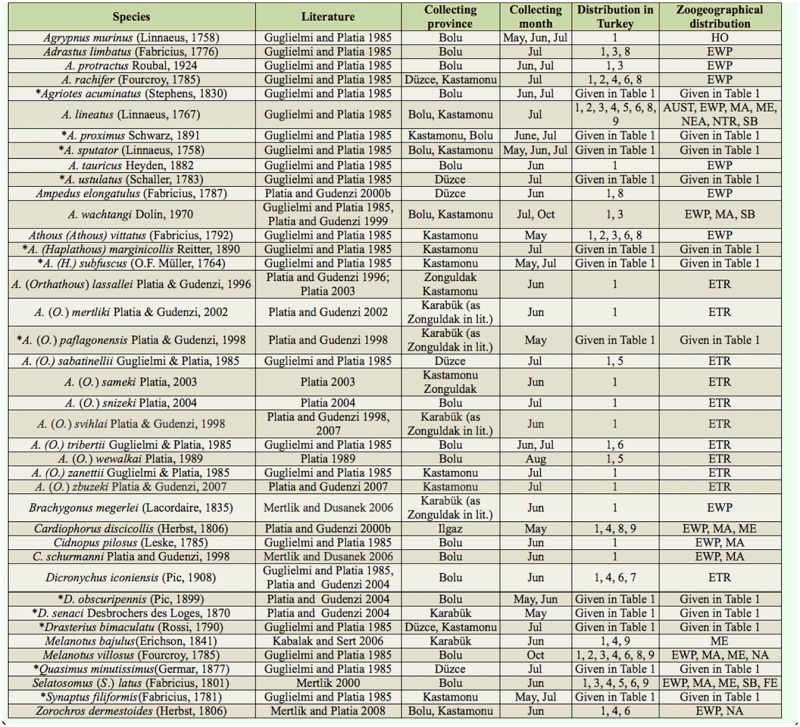
Months of collection and distribution of species within the research area as reported in the literature. **May:** May, **Jun:** June, **Jul:** July, **Aug:** August, **Oct:** October. **Distribution in Turkey: I:** Western Black Sea Region, **2:** Middle Black Sea Region, **3:** Eastern Black Sea Region, **4:** Central Anatolian Region, **5:** Marmara Region, **6:** Eastern Anatolian Region, **7:** South Eastern Anatolian Region, **8:** Aegean Region, **9:** Mediterranean Region (Dušánek and Mertlik 2007; Guglielmi and Platia 1985; Gülperçin and Tezcan 2009 and 2010; Kabalak 2010; Kabalak and Sert 2005, 2006, 2011a, 2011b; Kesdek et al. 2006; Lohse 1979; Mertlik 2000; Mertlik and Dušánek 2006; Mertlik and Platia 2008; Platia 1989, 2003; Platia and Gudenzi 1996, 1998, 2000a, 2000b, 2002, 2004; Platia and Schimmel 1991; Schimmel 1990; Sert and Kabalak 2011; Yüksel 1970); **Zoogeographical Distibution: AUST:** Australian, **ETR:** Endemic for Turkey, **EWP:** European part of the western Palaearctic, **FE:** Far East, **HO:** Holarctic, **MA:** Middle Asia, **ME:** Middle East, **NA:** North Africa, **NEA:** Nearctic, **NTR:** Neotropic, **PA:** Palearctic, **SB:** Siberia (Cate 2007; Mertlik and Platia 2008; Penev and Alekseev 1996; Platia 1994). An asterisk (*) indicates a species that was reported in the literature and also collected by authors.

**Table 3.**
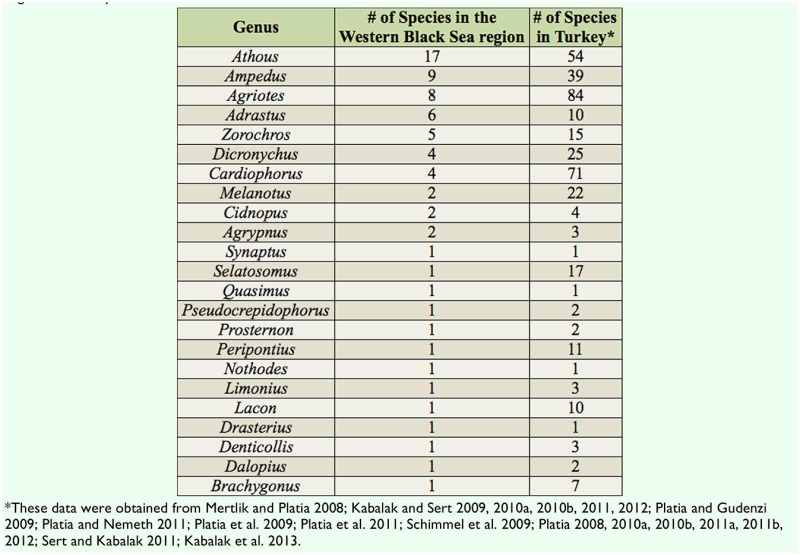
Comparison of the number of species in each genus of Elateridae between the Western Black Sea region and other regions of Turkey.

## Abbreviations

Aust: Australian region;
col.,: collector;
Dt-As,: decaying trees by aspirator;
ETr,: endemic to Turkey;
Ewp,: European part of the Western Palaearctic region;
Fe,: Far Eastern Asia;
Fhp-In,: ground herbaceous plants of the forest by insect net;
Ho,: Holarctic;
Hpfr-In,: herbaceous plants near fields and roads by insect net;
Hps-In,: herbaceous plants near streams by insect net;
Ma,: Middle Asia;
Me,: Middle East;
Na,: North Africa;
Nea,: Nearctic;
Ntr,: Neotropic;
Pa,: Palaearctic;
Sb,: Eastern and Western Siberian parts of Russia;
Tb-Ju,: trees and bushes by Japanese umbrella;
Uss-As,: under stones near streams by aspirator
